# A Questionnaire Survey of the Type of Support Required by *Yogo* Teachers to Effectively Manage Students Suspected of Having an Eating Disorder

**DOI:** 10.1186/s13030-016-0065-5

**Published:** 2016-05-09

**Authors:** Kaoru Seike, Hisashi Hanazawa, Toshiyuki Ohtani, Shizuo Takamiya, Ryoichi Sakuta, Michiko Nakazato

**Affiliations:** United Graduate School of Child Development, Osaka University, 2 Yamadaoka Suita-city, Osaka, Prefecture 565-0871 Japan; Research Center for Child Mental Development Chiba University, 1-8-1 Inohana, Chuo-ku, Chiba-city, Chiba Prefecture 260-8670 Japan; Faculty of Education, Chiba University, 1-33 Yayoi, Inage-ku, Chiba-city, Chiba Prefecture 263-0022 Japan; Safety and Health Organization, Chiba University, 1-33 Yayoi, Inage-ku, Chiba-city, Chiba Prefecture 263-0022 Japan; Department of Psychiatry, Nishi-Kobe Medical Center, 5-7-1 Koujidai, Nishi-ku, Kobe-city, Hyogo, Prefecture 651-2273 Japan; Center for Child Development and Psychosomatic Medicine, Dokkyo Medical University Koshigaya Hospital, 2-1-50 Minami-koshigaya, Koshigaya-city, Saitama Prefecture 343-0845, Japan

**Keywords:** Eating disorder, *Yogo* teacher, Surveillance, Support, DSM-5

## Abstract

**Background:**

Many studies have focused on the decreasing age of onset of eating disorders (EDs). Because school-age children with EDs are likely to suffer worse physical effects than adults, early detection and appropriate support are important. The cooperation of *Yogo* teachers is essential in helping these students to find appropriate care. To assist *Yogo* teachers, it is helpful to clarify the encounter rates (the proportion of *Yogo* teachers who have encountered ED students) and kinds of requested support (which *Yogo* teachers felt necessary to support ED students). There are no studies that have surveyed the prevalence rates of ED children by ED type as defined by the Diagnostic and Statistical Manual of Mental Disorders, 5th edition (DSM-5), nor were we able to find any quantitative study surveying the kinds of support *Yogo* teachers feel helpful to support ED students.

**Methods:**

A questionnaire survey was administered to 655 *Yogo* teachers working at elementary/junior high/senior high/special needs schools in Chiba Prefecture. The questionnaire asked if the respondents had encountered students with each of the ED types described in DSM-5 (anorexia nervosa (AN), bulimia nervosa (BN), binge eating disorder (BED), avoidant/restrictive food intake disorder (ARFID), and other types of EDs (Others)), and the kinds of support they felt necessary to support these students. The encounter rates and the kinds of requested were obtained and compared, taking their confidence intervals into consideration.

**Results:**

The encounter rates for AN, BN, BED, ARFID, and Others were 48.4, 14.0, 8.4, 10.7, and 4.6 %, respectively. When classified by school type, AN, BN, BED, and ARFID had their highest encounter rates in senior high schools. Special needs schools had the highest rate for Others. The support most required for all ED types was “a list of medical/consultation institutions.”

**Conclusions:**

Our results have clarified how to support *Yogo* teachers in the early detection and support of ED students. We found that the encounter rate of AN was the highest, and that it is effective to offer “a list of medical/consultation institutions” to junior and senior high schools where the encounter rates for AN are high.

## Background

Eating disorders (EDs) can be thought of as abnormalities in eating behavior formed by close interactions among psychological, physical and mental factors [1–4]. Among EDs, anorexia nervosa (AN) is usually accompanied by a sudden loss of weight, as well as other kinds of mental diseases and behavioral disorders, and it can be chronic and severe [5–8]. Its mortality rate is extremely high, at 7 % in Japan. With respect to age, AN prevails among teens, while bulimia nervosa (BN) is more common among people in their twenties, and the proportions of teens in the estimated onset ages of EDs are increasing yearly.

There have been many studies focusing on the lowering age of onset of EDs outside Japan [9–13]. Some have examined the prevalence rates of early onset EDs (EOEDs) in children aged from 5 to 13 [14], the prevalence rates by ED type in students aged 10 and up [15], lifelong prevalence rates and onset ages by ED type [16], and early signs of ED symptoms in nine-year-olds [17].

However, as epidemiological surveys of medical institutions could undercount these prevalence rates, actual condition surveys of schools and communities are needed. Thus, there have been a number of recent surveys of *Yogo* teachers in Japan [18–21].

*Yogo* teachers are unique teachers in Japan. They measure height and weight, and review medical records for each student. They not only take care of students like school nurses, but are also in charge of health education. They stay at school on weekdays, teach courses of adolescent health and have contact with parents. They watch both the mental and physical health of students through body measurements, daily health observation and information from other teachers or students. As they are not nurses, only some of them have nurse-licenses [18]. They play the role of gatekeepers screening out students that may have diseases and refer them to school physicians.

Many of these surveys have also noted the lowering age of onset of EDs: one study reports a third grader with AN, and another found that AN prevails among younger students than previously thought.

However, there have been few epidemiological surveys to date on the actual condition of EDs in elementary schools. Students with EDs are likely to suffer worse physical effects than adults. Therefore, it is crucial to identify and support ED students as early as possible, beginning even at the level of elementary school.

Although school physicians in compliance with Japanese law do medical check-ups and recommend that students suspected of having EDs consult medical institutions, ED students (especially those with AN) strongly resist seeking treatment [22]. This suggests that it is crucial to obtain help from *Yogo* teachers in leading ED students to consultation.

In that respect, *Yogo* teachers are in a very advantageous position for the early detection and support of ED students, since they regularly measure all students’ bodies and oversee the daily health of the whole school. One previous study highlighted that general members of the school staff are in the best position for identifying early signs of EDs and supporting ED students [23].

In order to improve the present situation, in which one half to one third of AN students do not visit a medical institution [18], it is necessary for *Yogo* teachers to be able to offer effective methods of support to ED students. However, *Yogo* teachers generally have a heavy work-load, as they not only take care of the mental and physical health of the students, but also teach courses of adolescent health and liaise with parents. Therefore, this leaves little time to support ED students, and support for the *Yogo* teachers themselves is needed.

To examine this situation, we surveyed encounter rates and needs. The “encounter rate” is defined as the proportion of *Yogo* teachers out of all *Yogo* teachers who have encountered ED students. By surveying this rate by school and ED type, effective support can be appropriately implemented (e.g., extra support for school types with a high encounter rate). We used an encounter rate instead of a prevalence rate, since what we wanted was not “type of EDs diagnosed by *Yogo* teachers” but “type of EDs doubted by *Yogo* teachers.” Moreover, the prevalence rate not only tends to appear lower than actual, but also is hard to obtain (as it is difficult for medical institutions to gain access to schools in Japan).

There have been many surveys of prevalence rates of EDs in students [15, 16, 18–20], but to the best of our knowledge, there have been no surveys to date on encounter rates among *Yogo* teachers. Moreover, previous studies on prevalence rates were based on DSM-IV diagnosis criteria, and rates by ED-type based on the newer criteria described in DSM-5 have not yet been surveyed. The ED types described in DSM-5 are AN, BN, binge eating disorder (BED), avoidant/restrictive food intake disorder (ARFID), and other EDs such as rumination disorder or pica (Others).

DSM diagnosis criteria of the American Psychiatric Association were revised in 2013, and “Eating Disorders” and “Feeding and Eating Disorders in Infancy or Early Childhood” in DSM-IV were integrated into a new diagnosis category “Eating Behavior Disorders and Eating Disorders.” This can be attributed to the fact that there was no need at the time to separate infancy or early childhood from other ages, as it was widely recognized that EDs, previously believed to emerge mainly in infancy or early childhood, actually emerged at later ages. However, the prevalence rates of EDs are reported to be higher in DSM-5 than in DSM-IV-TR [24, 25] because DSM-5 has increased the number of symptom names and offers more flexible diagnostic criteria [26–30]. Furthermore, one study [31] reports that ARFID prevails among younger students than AN or BN. These facts indicate that a new survey by ED type based on DSM-5 is necessary.

It is also necessary to clarify the needs of *Yogo* teachers; i.e., what kind of support they require to support ED students. However, while there have been qualitative studies that survey their needs, to the best of our knowledge, there has not yet been a quantitative one.

Therefore, the objective of the present study was to gather fundamental data that would be effective in supporting *Yogo* teachers in their early detection and support of ED students. More specifically, we carried out questionnaire surveys of *Yogo* teachers at several different levels of school to determine the prevalence of each DSM-5 ED type, and clarified the proportion of *Yogo* teachers who had encountered ED students (encounter rate), and the kinds of support they needed to effectively support ED students (requested support).

## Methods

### Sample selection

The subjects were *Yogo* teachers working at elementary, junior high, senior high, and special needs schools in Chiba Prefecture, Japan. Chiba Prefecture is located in the national capital region, and has Chiba City (one of the ordinance-designated cities, with population of more than half million) and Narita International Airport. Its population is 6.2 million, which came 6th in Japanese 47 prefectures.

### Ethics statement

The study was approved by the Ethics Committee of Chiba University. We explained our research to the educational committees of Chiba Prefecture and cities, school principals, the head of the *Yogo* teacher association, and the *Yogo* teachers through both written and oral descriptions. All participants gave their written informed consent. The questionnaire was anonymous and self-completed, and included the statement that all responses were entirely voluntary.

### Study procedures

The questionnaire asked about the demographic characteristics of the *Yogo* teachers, the features of their schools, their rates of encounter of ED students, their needs for support for those students. It also included an explanation of the DSM-5 categories and criteria, and the opportunity for the respondent to freely write his or her impressions of the survey, requests, etc. The question items were created by the Early Finding Working Group Committee. The demographic items included age, gender, nursing experience, years of experience as a *Yogo* teacher, and school type (elementary, junior high, senior high, or special needs). School feature items included gender type (boys’, girls’, or coeducational school), number of students, and location of the school. Encounter rate questions appeared in the yes/no format and asked if the respondent had encountered ED students by ED type. *Yogo* teachers were also asked how necessary they considered eight different kinds of support (very necessary, rather necessary, not very necessary, not necessary).

The questionnaires were distributed to 1,272 *Yogo* teachers of the specified types of schools in Chiba Prefecture during a Chiba Prefecture *Yogo* teacher seminar held in January of 2015, and were completed and collected on the spot (1272 covered 78.8 % of 1615, the total number of Yogo teachers of Chiba Prefecture).

We then carried out the following analyses:

1. A calculation of encounter rates for all ED types by dividing the number of *Yogo* teachers who had encountered each type of ED student by the total number of *Yogo* teachers who completed the survey. Note that the encounter rate is not a prevalence rate, but a proportion of *Yogo* teachers who had experience of taking care of or meeting students with EDs.
2. List kinds of requested support for all ED types by descending order of the number of *Yogo* teachers answering “very necessary” in each category.

Note that the number of all *Yogo* teachers included missing values. The statistical analysis software SPSS Ver. 21.0 (IBM, Tokyo, Japan) was used for all analyses.

## Results

### Demographics of the participants

From the 1,272 *Yogo* teachers to whom questionnaires were distributed, 655 responses were obtained (effective response rate 51.5 %). The appropriateness of this sample size was examined using a 95 % confidence interval (CI) of the encounter rate, which can be expressed as [*p*’ – 1.96√(*pq*/*n*), *p*’ + 1.96√(*pq*/*n*)] where *n* is the sample size, *p*’ is the sample proportion, *p* is the population proportion and *q* is *1*-*p*. When the interval is set within 5 %, the minimum required sample size is calculated as 385 (1.96√(*pq*/*n*) < 1.96√(.5*.5/*n*) < .05, *n* > {1.96*√(.5*.5/.05)}^2^ = 384.16). Furthermore, previous studies on *Yogo* teachers had sample sizes of 150 [20] and 391 [21]; thus, the present sample size of 655 was judged to be sufficient.

The demographic characteristics of the responding *Yogo* teachers are shown in Table 1. All were female, and 62.0 % were in their forties or older. 93.6 % lacked nursing experience, and 51.2 % had worked as a *Yogo* teacher for at least 20 years. 59.4 % worked at an elementary school, 28.9 % at a junior high school, 9.3 at a senior high school, and 2.4 % at a special needs school.

**Table 1:** Demographic Characteristics of *Yogo* teachers

		*n*	%
Gender	Male	0	0.0
	Female	655	100.0
	Missing	0	-
Age	20–29	133	20.3
	30–39	116	17.7
	40–49	197	30.1
	50–59	209	31.9
	Missing	0	-
Nursing Experience	yes	42	6.4
	no	613	93.6
	Missing	0	-
Years of Experience	1–5	129	20.0
	6–10	80	12.4
	11–20	105	16.3
	20–38	330	51.2
	Missing	11	-
School Type	Elementary school	389	59.4
	Junior high school	189	28.9
	Senior high school	61	9.3
	Special needs school	16	2.4
	Missing	0	-

The features of the schools are shown in Table 2. All schools were coeducational. Most elementary and junior high schools had 201 ~ 400 students and were located in local core cities. Most senior high schools had 801 ~ 1000 students and were located in suburbs.

**Table 2:** Features of Schools where *Yogo* teachers worked

		Elementary School (*N*=389)		Junior High School (*N*=189)		Senior High School (*N*=61)		Special Needs School (*N*=16)		Total	
		*n*	%	*n*	%	*n*	%	*n*	%	*n*	%
Gender Type	Boy’s School	0	0.0	0	0.0	0	0.0	0	0.0	0	0.0
	Girl's School	0	0.0	0	0.0	0	0.0	0	0.0	0	0.0
	Coeducation School	389	100.0	189	100.0	61	100.0	16	100.0	655	100.0
	Missing	0	-	0	-	0	-	0	-	0	-
Number of Students	1~60	25	6.6	5	2.8	1	1.7	1	6.7	32	5.0
	61~200	83	21.9	31	17.1	3	5.2	11	73.3	128	20.2
	201~400	101	26.6	62	34.3	5	8.6	3	20.0	171	27.0
	401~600	72	19.0	45	24.9	10	17.2	0	0.0	127	20.1
	601~800	65	17.2	20	11.0	11	19.0	0	0.0	96	15.2
	801~1000	25	6.6	15	8.3	22	37.9	0	0.0	62	9.8
	1001 +	8	2.1	3	1.7	6	10.3	0	0.0	17	2.7
	Missing	10	-	8	-	3	-	1	-	22	-
Location	Metropolis	51	14.0	22	12.7	14	25.9	3	21.4	90	14.9
	Suburbs	112	30.9	47	27.2	18	33.3	3	21.4	180	29.8
	Local Core Cities	137	37.7	78	45.1	16	29.6	7	50.0	238	39.4
	Local Towns or Villages	61	16.8	26	15.0	5	9.3	1	7.1	93	15.4
	Other	2	0.6	0	0.0	1	1.9	0	0.0	3	0.5
	Missing	26	-	16	-	7	-	2	-	41	-

Metropolis: Ordinance-designated city (Chiba City)

Suburbs: Cities, towns and villages surrounding Chiba City

Local Core Cities: Cities not located in Chiba City or its suburbs

Local Towns or Villages: Towns or villages not located in Chiba City or its suburbs

### Statistical analyses

**Fig. 1:**
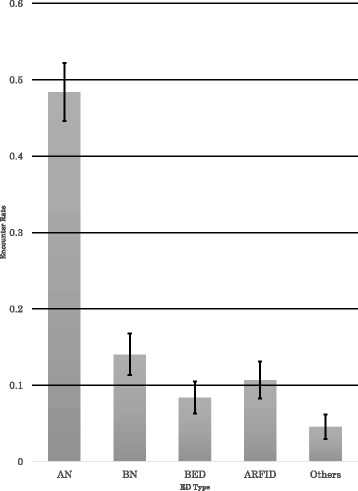
Encounter Rates by ED Type

Table 3 and Fig. 1 show encounter rates by ED type. With all school types combined, the rate of AN was highest at 48.4 %, followed by BN at 14.0 %, and then ARFID, BED and Others at 10.7, 8.4, and 4.6 %, respectively. Note that the order of BN > ARFID may be switched as their CIs overlapped (the CIs of BN and ARFID were 11.4 ~ 16.7 and 8.3 ~ 13.1 %, respectively).

**Table 3:** Encounter Rates by ED Type

ED Type	*n*	Encounter rate (95 % CI)
AN	317	0.484 (0.446–0.522)
BN	92	0.140 (0.114–0.167)
BED	55	0.084 (0.063–0.105)
ARFID	70	0.107 (0.083–0.131)
Others	30	0.046 (0.030–0.062)

n: number of school nurses, N=655

Table 4 shows the encounter rates by ED and school type. For AN, the encounter rate was highest in senior high school at 80.3, and junior high school came next at 67.7 %. For BN, senior high school was highest at 50.8 %. For BED, senior high school was again highest at 27.9 %, followed by special needs school and junior high school at 12.5 and 11.1 %, respectively. For ARFID, senior high school was highest at 14.8 %, followed by junior high school, elementary school and special needs school at 11.1, 10.0, and 6.3 %, respectively. For Others, special needs school was highest at 31.3 %.

**Table 4:** Encounter Rates by ED and School Type

ED Type	School Type	*n*	Encounter rate (95 % CI)
AN	Elementary school	134	0.344 (0.297–0.392)
	Junior high school	128	0.677 (0.611–0.744)
	Senior high school	49	0.803 (0.704–0.903)
	Special needs school	6	0.375 (0.138–0.612)
BN	Elementary school	27	0.069 (0.044–0.095)
	Junior high school	31	0.164 (0.111–0.217)
	Senior high school	31	0.508 (0.383–0.634)
	Special needs school	3	0.188 (0.000–0.379)
BED	Elementary school	15	0.039 (0.019–0.058)
	Junior high school	21	0.111 (0.066–0.156)
	Senior high school	17	0.279 (0.166–0.391)
	Special needs school	2	0.125 (0.000–0.287)
ARFID	Elementary school	39	0.100 (0.070–0.130)
	Junior high school	21	0.111 (0.066–0.156)
	Senior high school	9	0.148 (0.059–0.237)
	Special needs school	1	0.063 (0.000–0.181)
Others	Elementary school	15	0.039 (0.019–0.058)
	Junior high school	9	0.048 (0.017–0.078)
	Senior high school	1	0.016 (0.000–0.048)
	Special needs school	5	0.313 (0.085–0.540)

n: number of school nurses. Total number of school nurses at each school type: elementary school, 389; junior high school, 189; senior high school, 61; special needs school, 16

Table 5 shows the requested support by the descending order of the number of *Yogo* teachers who had encountered ED students (by ED type) and felt that the listed support was “very necessary.” The type of support considered most necessary was “a list of medical/consultation institutions” for all ED types at 66.7 ~ 85.5 %. For Others, note that “a list of medical/consultation institutions” may be switched with “education of teachers” or “advice from medical/consultation institutions” as their CIs overlapped.

**Table 5:** Support Requested by *Yogo* teachers by ED Type

Support required	AN (*N*=317)		BN (*N*=92)		BED (*N*=55)		ARFID (*N*=70)		Others (*N*=30)	
	*n*	Encounter rate (95 % CI)	*n*	Encounter rate (95 % CI)	*n*	Encounter rate (95 % CI)	*n*	Encounter rate (95 % CI)	*n*	Encounter rate (95 % CI)
List of medical/consultation institutions	248	0.78 (0.737–0.828)	75	0.82 (0.736–0.895)	47	0.86 (0.761–0.948)	50	0.71 (0.608–0.820)	20	0.67 (0.498–0.835)
Manual for supporting ED students	146	0.46 (0.406–0.515)	44	0.48 (0.376–0.580)	29	0.53 (0.395–0.659)	28	0.4 (0.285–0.515)	10	0.33 (0.165–0.502)
Guidelines for cooperating with medical institutions	143	0.45 (0.396–0.506)	40	0.44 (0.333–0.536)	24	0.44 (0.305–0.567)	23	0.33 (0.219–0.439)	10	0.33 (0.165–0.502)
Advice from medical/consultation institutions	157	0.5 (0.440–0.550)	51	0.55 (0.453–0.656)	29	0.53 (0.395–0.659)	32	0.46 (0.340–0.574)	12	0.4 (0.225–0.575)
Education of students	111	0.35 (0.298–0.403)	33	0.36 (0.261–0.457)	25	0.46 (0.323–0.586)	22	0.31 (0.206–0.423)	8	0.27 (0.108–0.425)
Education of guardians	131	0.41 (0.359–0.467)	39	0.42 (0.323–0.525)	27	0.49 (0.359–0.623)	27	0.39 (0.272–0.500)	10	0.33 (0.165–0.502)
Education of teachers	150	0.47 (0.418–0.528)	42	0.46 (0.355–0.558)	28	0.51 (0.377–0.641)	28	0.4 (0.285–0.515)	14	0.47 (0.288–0.645)
Education of school club advisors	133	0.42 (0.365–0.474)	39	0.42 (0.323–0.525)	23	0.42 (0.288–0.549)	22	0.31 (0.206–0.423)	8	0.27 (0.108–0.425)

*n* number of school nurses that considered the given type of support “very necessary”

Table 6 shows requested support by school type. The support most requested by *Yogo* teachers of elementary, junior high, and senior high schools was “a list of medical/consultation institutions” at 73.5, 82.4, and 82.2 % respectively. The support most requested by special needs schools was “education of teachers” at 64.7 %, followed by “a list of medical/consultation institutions” and “advice from medical/consultation institutions” at 58.8 and 58.8 %, respectively. Note that this order may be reversed as CIs overlapped.

**Table 6:** Support Requested by *Yogo* teachers by School Type

Support required	Elementary school (*N*=230)		Junior high school (*N*=210)		Senior high school (*N*=107)		Special needs school (*N*=17)	
	*n*	Encounter rate (95 % CI)	*n*	Encounter rate (95 % CI)	*n*	Encounter rate (95 % CI)	*n*	Encounter rate (95 % CI)
List of medical/consultation institutions	169	0.735 (0.677–0.792)	173	0.824 (0.772–0.875)	88	0.822 (0.750–0.895)	10	0.588 0.354–0.822)
Manual for supporting ED students	102	0.443 0.379–0.508)	94	0.448 (0.380–0.515)	56	0.523 (0.429–0.618)	5	0.294 (0.078–0.511)
Guidelines for cooperating with medical institutions	90	0.391 (0.328–0.454)	82	0.390 (0.324–0.456)	63	0.589 (0.496–0.682)	5	0.294 (0.078–0.511)
Advice from medical/consultation institutions	98	0.426 (0.362–0.490)	111	0.529 (0.461–0.596)	62	0.579 (0.486–0.673)	10	0.588 (0.354–0.822)
Education of students	73	0.317 (0.257–0.378)	65	0.309 (0.247–0.372)	54	0.505 (0.410–0.599)	7	0.412 (0.178–0.646)
Education of guardians	91	0.396 (0.332–0.459)	86	0.410 0.343–0.476)	50	0.467 (0.373–0.562)	7	0.412 (0.178–0.646)
Education of teachers	92	0.400 (0.337–0.463)	110	0.524 (0.456–0.591)	49	0.458 (0.364–0.552)	11	0.647 (0.420–0.874)
Education of school club advisors	77	0.335 (0.274–0.396)	97	0.462 (0.394–0.530)	46	0.430 (0.336–0.524)	5	0.294 (0.078–0.511)

*n* number of school nurses that considered the given type of support “very necessary”

### Free-description

A free-description section contained some valuable writings regarding tools or information for screening ED students. A number of *Yogo* teachers reported using growth curves, or information from other teachers, students and parents (such as their seeing of suspicious students’ vomiting) for that purpose.

Regarding screening AN students, *Yogo *teachers reported comments such as “I used growth curves,” “I wish there was a method of finding EDs from growth curves promptly.” Regarding BNs, *Yogo* teachers reported “I saw some students vomit after lunch.” “I heard information of students’ vomiting from other students or teachers.” Regarding ARFIDs, “I was asked to respond to students avoiding taking lunch,” “ARFID is more common among boys.”

## Discussion

The objective of the present study was to gather fundamental data to clarify what types of support might be effective in aiding *Yogo* teachers with the early detection and support of ED students. Specifically, we conducted a questionnaire survey of *Yogo* teachers working at elementary, junior high, senior high, and special needs schools, and clarified the proportion of *Yogo* teachers who had encountered ED students (encounter rate), and the kinds of support they felt they needed to support those students (support). EDs were divided into 5 different categories based on DSM-5 criteria.

The ED type with the highest encounter rate was AN. While some previous studies on students have reported that the prevalence of Eating Disorder Not Otherwise Specified (EDNOS) was higher than that of AN [15, 16] outside Japan, other research on *Yogo* teachers has found that AN is more common than BN in Japan. This difference may be explained by the fact that Japanese law required *Yogo* teachers to draw growth curves, which enabled them to detect ANs easily as described in a free-description section. BMI is frequently used in surveys of EDs [32–36], but is not common in Japan. As *Yogo* teachers are busy with not only taking care of students but also teaching classes, and do not have much time to calculate them. Instead, they prefer to use growth curves.

The present results show that as many as half (48.4 %) of *Yogo* teachers have encountered AN students. Thus, it may be effective to assist *Yogo* teachers with support intended primarily for students with AN.

England has General Practitioners (GPs) and Child and Adolescent Mental Health Services (CAMHS) outside school, as well as guidelines and a flowchart to help ED students find appropriate medical institutions in school [37]. In contrast, Japan has no concrete support for ED students in school. The present study revealed that the most necessary support for *Yogo* teachers who had encountered students with AN was “a list of medical/consultation institutions.” Moreover, a previous study that asked *Yogo* teachers what they wanted to know about EDs reported that “a way of cooperating with medical institutions” was highly needed. These facts indicate that it is necessary to offer *Yogo* teachers, especially those dealing with AN, “a medical/consultation institution list,” which would connect schools and medical institutions.

Next, the encounter rates of AN were compared by school type in order to examine which types of school should be supported as a first priority. A questionnaire survey of *Yogo* teachers based on DSM-IV reported that the numbers of students reported as “too thin” and “eat and vomit” in senior high school were higher than those in junior high school. The present results also showed that the encounter rate in senior high school was higher than that in junior high school. These facts indicate that it may be effective to offer “a list of medical/consultation institutions” to senior high schools when supporting AN students.

BN had the second highest encounter rate at 14.0 %. This may be attributed to the fact that *Yogo* teachers often saw students vomit, or obtained information of vomiting students from other students or teachers as mentioned in a free description section. For the support needed, *Yogo* teachers again reported that the most needed was “a list of medical/consultation institutions.” The rate was highest in senior high schools.

Similarly, the support most requested by *Yogo* teachers who had encountered students with ARFID was “a list of medical/consultation institutions.” The encounter rates for all school types were at the same level. One previous report found that ARFID is “a phenomenon seen not only in infants less than seven years old or in early childhood, but also in a wider range of ages such as school age or prepuberty” [31]. In addition, comments in a free description section revealed that *Yogo* teachers frequently saw ARFID students avoid taking lunch in school, and were required to respond their likes and dislikes of food by parents or teachers. These facts imply that it is necessary to support ARFID from elementary school age, and assist *Yogo* teachers with a list of medical/consultation institutions and support for handling food dislikes.

It is essential to offer ED support, as it is considered to be highly needed by *Yogo* teachers at each type of school, and the type of support most overwhelmingly requested by *Yogo* teachers who had encountered students with EDs was “a list of medical/consultation institutions.” A previous study states that the lowering age of onset of EDs and prolongation are major problems of EDs. We therefore believe that it is important to offer “a list of medical/consultation institutions” at the elementary school level, and continue to provide support to ED students throughout their school years.

The types of support required by *Yogo* teachers of special needs schools who had encountered EDs were “education of teachers,” “a list of medical/consultation institutions” and “advice from medical/consultation institutions.” Special needs schools are specialized schools that accept students with developmental disorders. Some studies have noted that students with developmental disorders tend to develop EDs [38, 39]. Thus, it may be useful to offer the support requested by *Yogo* teachers of special needs schools.

Lastly, we discuss the validity of using encounter rate. We obtained the proportion of *Yogo* teachers who had encountered EDs and not the actual proportion of ED students, prevalence. In some cases, *Yogo* teachers may not be able to detect EDs or differentiate ED types, either because they are not familiar with them or ED students avoid visiting *Yogo* teachers. This suggests that using the prevalence rate may be more appropriate.

However, the prevalence is not lack of flaws. It is hard to obtain since it is difficult for medical institutions to obtain access to schools in Japan. School physicians may do so, but they seldom go to school and their surveys may not be timely. Comparing with them, *Yogo* teachers stay schools on daily basis, evaluating the health condition of students real time.

Moreover, as our purpose was to clarify the needs of *Yogo* teacher who deal with EDs, what we needed may not necessarily be “an accurate proportion of ED students diagnosed by doctors”, and “a proportion of ED students doubted by *Yogo* teachers” was enough for the purpose. Therefore, we concluded that using the encounter rate was valid in this survey.

### Limitations and recommendations

One of the limitations of the present study is that the sample might be biased as all participants were only selected from only one prefecture, Chiba Prefecture. In addition, the sample sizes of the special needs schools and Others were small, which made CIs long and estimation errors large. By increasing the sample size and selecting participants from various prefectures, it will be possible to compare the encounter and prevalence rates of multiple prefectures. We believe that this would lead to better support for *Yogo* teachers in finding and supporting ED students.

Moreover, as we did not ask *Yogo* teachers to calculate BMIs or draw growth charts for screening ED students, our results may be regarded as lacking in objectivity. However as, our purpose was not to investigate the objective prevalence, but to clarify the needs of Yogo teachers in supporting EDs, asking them to use such tools seems unnecessary.

## Conclusion

The present study was a questionnaire survey of *Yogo* teachers at elementary, junior high, senior high, and special needs schools in Chiba Prefecture, Japan. We calculated the encounter rates and the requested support by school and by ED type. Our results showed that the order of encounter rates was AN > BN > ARFID, and that it might be effective to offer a “medical/consultation institution list” for the early detection and support of ED students for all ED types. By individual ED type, it was found that it might be effective to offer support to *Yogo* teachers in senior and junior high schools for AN, in senior high schools for BN, and in all school types for ARFID.

## Abbreviations

AN: anorexia nervosa
ARFID: avoidant/restrictive food intake disorder
BED: binge eating disorder
BN: bulimia nervosa
CI: confidence interval
ED: eating disorder
EDNOS: Eating Disorder Not Otherwise Specified.
EOED: early onset EDs
Others: pica rumination disorder, etc

## References

[1] Micali N, Stahl D, Treasure J, Simonoff E (2014). Childhood psychopathology in children of women with eating disorders: understanding risk mechanisms. J Child Psychol Psychiatry.

[2] Mann AP, Accurso EC, Stiles-Shields C, Capra L, Labuschagne Z, Karnik NS, Le Grange D (2014). Factors associated with substance use in adolescents with eating disorders. J Adolesc Health.

[3] Harrison C, Mond J, Bentley C, Gratwick-Sarll K, Rieger E, Rodgers B (2014). Loss of control eating with and without the undue influence of weight or shape on self-evaluation: evidence from an adolescent population. J Eat Disord.

[4] Rohde P, Stice E, Marti CN (2015). Development and predictive effects of eating disorder risk factors during adolescence: Implications for prevention efforts. Int J Eat Disord.

[5] Lang K, Lloyd S, Khondoker M, Simic M, Treasure J, Tchanturia K (2015). Do children and adolescents with anorexia nervosa display an inefficient cognitive processing style?. PLoS One.

[6] Neumärker KJ (1997). Mortality and sudden death in anorexia nervosa. Int J Eat Disord.

[7] Papadopoulos FC, Ekbom A, Brandt L, Ekselius L (2009). Excess mortality, causes of death and prognostic factors in anorexia nervosa. Br J Psychiatry.

[8] Lang K, Dapelo MM, Khondoker M, Morris R, Surguladze S, Treasure J, Tchanturia K (2015). Exploring emotion recognition in adults and adolescents with anorexia nervosa using a body motion paradigm. Eur Eat Disord Rev.

[9] Kurz S, van Dyck Z, Dremmel D, Munsch S, Hilbert A (2015). Early-onset restrictive eating disturbances in primary school boys and girls. Eur Child Adolesc Psychiatry.

[10] Cardi V, Corfield F, Leppanen J, Rhind C, Deriziotis S, Hadjimichalis A, Hibbs R, Micali N, Treasure J (2014). Emotional processing of infants displays in eating disorders. PLoS One.

[11] Kothari R, Rosinska M, Treasure J, Micali N (2014). The early cognitive development of children at high risk of developing an eating disorder. Eur Eat Disord Rev.

[12] Kelly NR, Shank LM, Bakalar JL, Tanofsky-Kraff M (2014). Pediatric feeding and eating disorders: current state of diagnosis and treatment. Curr Psychiatry Rep.

[13] Nicely TA, Lane-Loney S, Masciulli E, Hollenbeak CS, Ornstein RM (2014). Prevalence and characteristics of avoidant/restrictive food intake disorder in a cohort of young patients in day treatment for eating disorders. J Eat Disord.

[14] Madden S, Morris A, Zurynski YA, Kohn M, Elliot EJ (2009). Burden of eating disorders in 5-13-year-old children in Australia. Med J Aust.

[15] Micali N, Hagberg KW, Petersen I, Treasure JL (2013). The incidence of eating disorders in the UK in 2000–2009: findings from the General Practice Research Database. BMJ Open.

[16] Swanson SA, Crow SJ, Grange DL, Swendsen J, Merikangas KR (2011). Prevalence and Correlates of eating disorders in adolescents. Arch Gen Psychiatry.

[17] Parkinson KN, Drewett RF, Couteur AS, Adamson AJ (2012). The gateshead millennium study core team: earlier predictors of eating disorder symptoms in 9-year-old children. A longitudinal study. Appetite.

[18] Hotta M, Horikawa R, Mabe H, Yokoyama S, Sugiyama E, Yonekawa T (2015). Epidemiology of anorexia nervosa in Japanese adolescents. BioPsychoSocial Medicine.

[19] Komaki G, Kachi Y (2005). Eight prefecture survey of school nurses inquiring about the risk factors, early detection methods and intervention for disordered eating among Japanese teenagers. Japanese Journal of Psychosomatic Medicine.

[20] Suzuki HM, Ohara T, Horikawa R, Ogawa Y (2013). The epidemiology survey of the anorexia nervosa by the questionnaire to teachers in charge of health education of high schools in Tokyo. Japanese Journal of Psychosomatic Internal Medicine.

[21] Karaki M, Takamiya S, Kawazoe A (2014). Journal of Japanese Society of Psychosomatic Pediatrics.

[22] Nakai Y (2012). Epidemiology of eating disorders. Journal of Clinical and Experimental Medicine.

[23] Knightsmith P, Treasure J, Schmidt U (2013). Spotting and supporting eating disorders in school: recommendations from school staff. Health Educ Res.

[24] Allen KL, Ryrne SM, Oddy WH, Crosby RD (2013). DSM-IV-TR and DSM-5 eating disorders in adolescents: prevalence, stability, and psychosocial correlates in a population-based sample of male and female adolescents. J Abnorm Psychol.

[25] Birgegard A, Norring C, Clinton D (2012). DSM-IV versus DSM-5: Implementation of proposed DSM-5 criteria in a large naturalistic database. Int J Eating Disord.

[26] Goldschmidt AB, Accurso EC, O’Brien S, Kara Fitzpatrick K, Lock JD, Le Grange D (2016). The importance of loss of control while eating in adolescents with purging disorder. Int J Eat Disord.

[27] Norris ML, Katzman DK (2015). Change is never easy, but It is possible: reflections on avoidant/restrictive food intake disorder Two years after its introduction in the DSM-5. J Adolesc Health.

[28] Flament MF, Henderson K, Buchholz A, Obeid N, Nguyen HN, Birmingham M, Goldfield G (2015). Weight status and DSM-5 diagnoses of eating disorders in adolescents from the community. J Am Acad Child Adolesc Psychiatry.

[29] Mancuso SG, Newton JR, Bosanac P, Rossell SL, Nesci JB, Castle DJ (2015). Classification of eating disorders: comparison of relative prevalence rates using DSM-IV and DSM-5 criteria. Br J Psychiatry.

[30] Fisher M, Gonzalez M, Malizio J (2015). Eating disorders in adolescents: how does the DSM-5 change the diagnosis?. Int J Adolesc Med Health.

[31] Fisher MM, Rosen DS, Ornstein RM, Mammel KA, Katzman DK, Rome ES, Walsh BT (2014). Characteristics of avoidant/restrictive food intake disorder in children and adolescent :a “New disorder” in DSM-5. J Adolesc Health.

[32] Jones M, Taylor Lynch K, Kass AE, Burrows A, Williams J, Wilfley DE, Taylor CB (2014). Healthy weight regulation and eating disorder prevention in high school students: a universal and targeted Web-based intervention. J Med Internet Res.

[33] Ibrahim C, El-Kamary SS, Bailey J, St George DM (2014). Inaccurate weight perception is associated with extreme weight-management practices in U.S. high school students. J Pediatr Gastroenterol Nutr.

[34] Lopez V, Corona R, Halfond R (2013). Effects of gender, media influences, and traditional gender role orientation on disordered eating and appearance concerns among Latino adolescents. J Adolesc.

[35] Hou F, Xu S, Zhao Y, Lu Q, Zhang S, Zu P, Sun Y, Su P, Tao F (2013). Effects of emotional symptoms and life stress on eating behaviors among adolescents. Appetite.

[36] Radmanović-Burgić M, Gavrić Z, Burgić S (2011). Eating attitudes in adolescent girls. Psychiatr Danub.

[37] NICE (2004). Eating disorders-core interventions in the treatment and management of anorexia nervosa, bulimia nervosa and related eating disorders.

[38] Kliebert ML, Tiger JH (2011). Direct and distal effects of noncontingent juice on rumination exhibited by a child with autism. J Appl Behav Anal.

[39] Gillberg C (1983). Are autism and anorexia nervosa related?. Br J Psychiatry.

